# Association of Hyperparathyroidism with Depression and Anxiety Among Chronic Hemodialysis Patients in the Al Baha Region, Kingdom of Saudi Arabia

**DOI:** 10.7759/cureus.57210

**Published:** 2024-03-29

**Authors:** Areej I. Alhazmi, Abdullah Mushra Alghamdi, Fahad S Alghamdi, Maathir N Alhumam, Mujahid Khalid Nasser Alghamdi, Ghayda A Alghamdi, Salman Ahmed S Jathmi

**Affiliations:** 1 Internal Medicine/Nephrology, Al Baha University, Al Baha, SAU; 2 College of Medicine, Al Baha University, Al Baha, SAU; 3 Family Medicine, Primary Health Care Centre, Ar Rass, SAU; 4 College of Medicine, Taibah University, Al-Madinah Al-Munawarah, SAU

**Keywords:** hemodialysis, hads, anxiety, depression, parathyroid hormone

## Abstract

Introduction

Anxiety and depression are prevalent psychological issues among hemodialysis patients, adversely affecting their well-being and treatment response. The study aims to identify the relationship between these mental health concerns and hyperparathyroidism in chronic hemodialysis patients from the Al Baha Region, Kingdom of Saudi Arabia.

Methods

This retrospective study included 143 chronic hemodialysis patients aged 18-85 years. Monthly laboratory records for parathyroid hormone (PTH) levels and the Hospital Anxiety and Depression Scale (HADS) for mental health assessment were utilized. Demographic information and the primary causes of end-stage renal disease were obtained through patient interviews. Statistical analyses, including chi-square tests, odds ratio, and significance tests, were performed to assess associations.

Results

Elevated PTH levels were associated with increased anxiety and depression in hemodialysis patients. Patients with PTH levels >400 pg/ml exhibited higher rates of abnormal HADS scores for anxiety and depression than those with PTH levels <400 pg/ml. Gender differences were evident, with women showing a higher predisposition to anxiety disorders and men having depression. Additionally, patients with PTH levels <150 pg/ml had a significantly higher proportion of the “normal” depression score than those with PTH levels >800 pg/ml.

Conclusion

The study underscores the association between hyperparathyroidism and adverse mental health outcomes in chronic hemodialysis patients. Maintaining optimal PTH levels plays a crucial role in mitigating anxiety and depression. Gender differences in mental health outcomes highlight the need for tailored interventions. Routine mental health assessments, utilizing tools such as the HADS, are important in the comprehensive care of hemodialysis patients.

## Introduction

Anxiety and depression are the most common psychological impacts among hemodialysis patients that affect their well-being and response to treatment [1]. Many studies showed a relationship between anxiety disorder and suicidal thoughts which raises the importance of psychiatric assessment and identification of the underlying causes [2,3]. Many scales were used to identify these major psychological illnesses, including the Beck Depression Inventory (BDI), Hamilton Anxiety Rating Scale (HARS), and Hospital Anxiety and Depression Scale (HADS). A cross-sectional survey that a Chinese third-class hospital applied showed that depression, skin itchiness, and bone discomfort all had high prevalence rates of 94.06%, 69.06%, and 77.81%, respectively [4]. Another study conducted in Recife, Brazil, that included 173 hemodialysis patients aged 60 years old and above, noted that 43.3% had depression symptoms [5].

Patients with chronic kidney disease (CKD) frequently experience secondary hyperparathyroidism, a symptom of CKD-mineral bone condition (CKD-MBD). This condition is associated with a low quality of life as a result of bone pain and skin itching that increase the likelihood of a relationship with depression [6,7]. A study stated that numerous laboratory variables, including hemoglobin, parathyroid hormone (PTH), vitamin D, C-reactive protein, and fibrinogen, were linked with the BDI and HARS [8]. However, a statistically significant link between the BDI and PTH was not discovered. In addition, another study concluded that anxiety was negatively associated with intact PTH (iPTH) levels [7]. The Dialysis Outcomes and Practice Patterns Study found that hemodialysis patients had a significant rate of depression and hypercalcemia were significantly correlated; even after adjusting for age, gender, and other variables, the correlation remained significant [3]. Also, they reported no relationship was noted between mental health and iPTH. They advise further studies to draw firm conclusions on the link between PTH and mental health in dialysis patients.

The HADS is a simple, reliable, and valid tool for identifying depression and anxiety, particularly in patients with comorbidities that require frequent hospital visits and admissions [9-12]. This study aims to determine the association between hyperparathyroidism and depression as well as anxiety among chronic hemodialysis patients in the Al Baha Region, Kingdom of Saudi Arabia, using the HADS scale. This study also aims to find whether gender difference plays a role and to identify the incidents of depression and anxiety among the study population.

## Materials and methods

Study design

This retrospective study involved 160 chronic hemodialysis patients. Their files were reviewed from June 2022 to July 2023 in the Al Baha Region, Saudi Arabia, using an online evaluation form and electronic medical records for PTH laboratory results. This study was conducted at four hemodialysis centers belonging to the Ministry of Health, distributed over four areas (Al Baha, Al Makhwah, Al Mandaq, and Al Aqiq). These centers were selected to ensure representation from different areas within the region. The Institutional Review Board of Al Baha University approved the study (approval number: REC/MED//BU-FM/2023/2 dated March 31, 2023)

Participants

The treatment group in this study included chronic hemodialysis patients from the selected centers who met the following criteria: patients who had been undergoing hemodialysis for at least six months, aged between 18 and 85 years, had never been diagnosed or treated for depression or anxiety, and had an elevated serum PTH level from their laboratory results with at least two results indicating above 400 pg/ml. The control group included patients who presented with two results of serum PTH below 400 pg/ml. Moreover, patients in both groups had to be able to provide their medical history. The exclusion criteria included pediatric patients, peritoneal dialysis patients, and those unable to give a history by themselves or refused to complete the questionnaire.

We selected 160 patients from the centers of which 17 were excluded from this study: five died during the data collection, seven refused to complete the questionnaire, and five were not able to provide information by themselves.

Data collection

Laboratory data for serum PTH results were collected by the researchers from the laboratory monthly records and electronic medical records at the hospitals. They reviewed and collected results for one year, focusing on each patient having two readings or more for PTH over six months or more.

The HADS was used in this study to detect anxiety and depression. This tool is a reliable and valid instrument for a hospital medical outpatient clinic, such as hemodialysis [13,14]. It included seven items for each subscale that range from 0 to 3. The estimated measurement of each subscale categorizes patients as normal, borderline, or abnormal.

Data collected via the questionnaire form included three parts: (1) demographic data (such as age and gender, in addition to the primary cause of renal disease), (2) identification of depression and anxiety using the HADS scale, and (3) the result of the iPTH level. Well-trained researchers collected these data through patient interviews. 

Data analysis

Demographic factors were calculated using frequencies and percentages. To measure the association, the odds ratio (OR) was calculated as it provides information about the strength of the relationship between the exposure and outcome, as well as compares the risks between the exposed group and a comparison group. The data were analyzed using IBM SPSS Statistics for Windows, Version 26.0 (Released 2019; IBM Corp., Armonk, New York, United States), and any significant findings or associations were reported.

## Results

**Figure 1:**
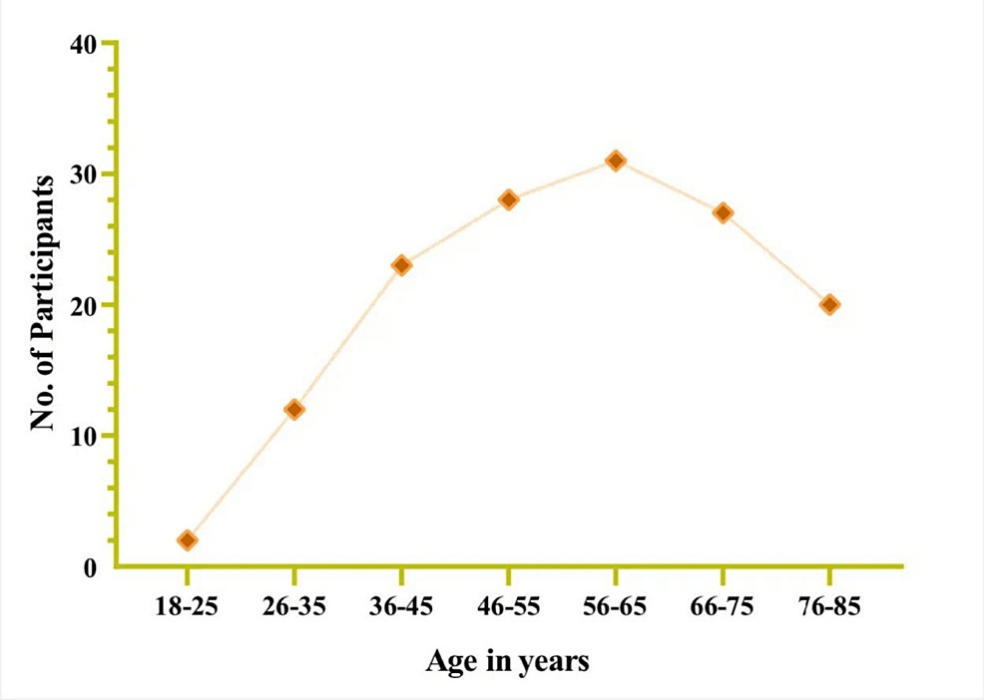
Distribution of number of participants as per their age ranges

Table 1 presents the demographic features of 143 participants in this study. All the participants were diagnosed with end-stage renal disease on hemodialysis. They were from four centers in the Al Baha Region, and the majority of them were from the Al Baha center (n = 67, 47%). The participants’ ages ranged between 18 and 85 years, with most aged 56-65 (n = 31, 21.7%). The lowest participant ages ranged between 18 and 25 years (n = 2, 1.4%) (Figure 1). Male patients totaled 89 (62.2%), whereas female patients numbered 54 (37.8%). The primary cause of end-stage renal disease was diabetes mellitus (n = 45, 31.5%) and hypertension (n = 43, 30%), amounting to two-thirds of the participants.

**Table 1:** Demographic features of the participants (N = 143) ESRD: end-stage renal disease

Variable	Number	Percentage
Age in Years		
18-25	2	1.4%
26-35	12	8.4%
36-45	23	16%
46-55	28	19.6%
56-65	31	21.7%
66-75	27	18.9%
76-85	20	14%
Gender		
Male	89	62.2%
Female	54	37.8%
Primary Cause of ESRD		
Diabetes mellitus	45	31.5%
Hypertension	43	30%
Medication	10	7%
Polycystic kidney disease	1	0.7%
Glomerulonephritis	3	2.1%
Urological problem	2	1.4%
Unknown	39	27.3%
Centers		
Al Baha	67	47%
Al Makhwah	39	27.3%
Al Mandaq	21	14.7%
Al Aqiq	16	11%

Analysis was done to diagnose anxiety and depression based on the HADS scores in patients with PTH levels above 400 pg/ml and those with PTH levels below 400 pg/ml. For depression and anxiety scores, we referred HADS-1, HADS-2, and HADS-3 to normal scores, borderline scores, and abnormal scores, with 0-7, 8-10, and 11-21 points, respectively.

Logistic regression analysis of the significant risk of elevated PTH revealed that in the case of depression, for individuals with PTH levels >400 pg/ml, 34, 12, and 16 cases were classified as abnormal, borderline, and normal, respectively. Compared with individuals with PTH levels <400 pg/ml, 19, 16, and 46 cases were classified as abnormal, borderline, and normal, respectively. The serum PTH level of the patients in the study group having PTH >400 pg/ml significantly increased. They were 3.47 times more likely to develop depression than patients in the control group with PTH <400 pg/ml (OR: 3.47, 95%CI 1.710-7.079, p = 0.01). Similarly, in the case of anxiety, for individuals with PTH levels >400 pg/ml, 17, 9, and 36 cases were classified as abnormal, borderline, and normal, respectively. Compared with individuals with PTH levels <400 pg/ml, 9, 7, and 65 cases were classified as abnormal, borderline, and normal, respectively. Patients in the study group were 2.934 times more subjected to the incidence of anxiety than the control group (OR: 2.934, 95%CI 1.394-6.175, p = 0.05) due to varying levels of serum PTH levels (Table 2). An analysis of the potential interplay of gender in the serum PTH level revealed no significant impact on the overall PTH levels of patients with p = 0.886.

**Table 2:** Logistic regression analysis for potential risk factors *Indicating statistically significant value (P ≤ 0.05) PTH: parathyroid hormone

Variable	Level of variable	Odds ratio	Confidence interval 95%	p-value
Depression				
Level of PTH	>400 pg/ml	3.47	1.710-7.079	0.01*
	<400 pg/ml			
Anxiety				
Level of PTH	>400 pg/ml	2.934	1.394-6.175	0.05*
	<400 pg/ml			

Table 3 shows the association of various risk factors with depression incidence. Overall, patients in the study group with serum PTH levels >400 pg/ml were more prone to the development of depression than the control group. Analysis of the male population with PTH levels >400 pg/ml showed that 24, 6, and 9 cases were abnormal, borderline, and normal. Comparing those having PTH levels <400 pg/ml, 10, 11, and 29 cases were classified as abnormal, borderline, and normal, respectively. The result was significant with an OR of 7.7333 (95%CI 2.7048-22.1101, p = 0.0001), comparing abnormal with normal value scores. For females with PTH levels >400 pg/ml, 10, 6, and 7 cases were abnormal, borderline, and normal. Comparing females having PTH levels <400 pg/ml, 9, 5, and 17 cases were classified as abnormal, borderline, and normal, respectively. This result was not significant (95%CI 0.7660-9.5062, p = 0.1223). In the population with PTH levels >800 pg/ml, 15, 3, and 6 cases were abnormal, borderline, and normal, respectively. When comparing individuals with PTH levels <150 pg/ml, 6, 5, and 13 cases were abnormal, borderline, and normal, respectively, with OR = 5.4167 (95%CI 1.3992-20.9691, p = 0.0144). Therefore, the serum PTH >800 pg/ml was significantly associated with depression.

**Table 3:** Association of various risk factors with depression *Indicating statistically significant value (P ≤ 0.05) PTH: parathyroid hormone; HADS: Hospital Anxiety and Depression Scale HADS-1, Normal scores 0-7 points; HADS-2, Borderline scores 8-10 points; HADS-3, Abnormal scores 11-21 points

Attribute	Depression			
	HADS-1	HADS-2	HADS-3	p-value
Male PTH > 400 pg/ml	9	6	24	0.0001*
Male PTH< 400 pg/ml	29	11	10	
Female PTH> 400 pg/ml	7	6	10	0.1223
Female PTH< 400 pg/ml	17	5	9	
PTH > 800 pg/ml	6	3	15	0.0144*
PTH < 150 pg/ml	13	5	6	

Risk factor analysis for anxiety in Table 4 reveals that the overall level of serum PTH >400 pg/ml had a significant impact on anxiety in the study group compared with the control group. Examining the male population with PTH levels >400 pg/ml, 3, 7, and 29 cases were abnormal, borderline, and normal, respectively. Compared with males having PTH levels <400/pg/ml, 1, 2, and 47 cases were classified as abnormal, borderline, and 47 normal, respectively, which was not significant (95% CI: 0.4826-48.9851, p = 0.1797). For females with PTH levels >400 pg/ml, 14, 2, and 7 cases were abnormal, borderline, and normal, respectively. Compared with females having PTH levels <400 pg/ml, 8, 5, and 18 cases were classified as abnormal, borderline, and normal, respectively. This result was significant, with OR = 4.5 for abnormal versus normal scores (95%CI 1.3132-15.4201, p = 0.0167). In the population with PTH levels >800 pg/ml, 6, 2, and 16 cases were abnormal, borderline, and normal, respectively. When compared with individuals with PTH levels <150 pg/ml, 3, 2, and 19 cases were abnormal, borderline, and normal, respectively, which was not significant (95%CI 0.5106-11.0478, p = 0.2701).

**Table 4:** Association of various risk factors with anxiety *Indicating statistically significant value (P ≤ 0.05) PTH: parathyroid hormone; HADS: Hospital Anxiety and Depression Scale HADS-1, Normal scores 0-7 points; HADS-2, Borderline scores 8-10 points; HADS-3, Abnormal scores 11-21 points

Attribute	Anxiety			
	HADS-1	HADS-2	HADS-3	p-value
Male PTH> 400 pg/ml	29	7	3	0.1797
Male PTH< 400 pg/ml	47	2	1	
Female PTH> 400 pg/ml	7	2	14	0.0167*
Female PTH< 400 pg/ml	18	5	8	
PTH > 800 pg/ml	16	2	6	0.2701
PTH < 150 pg/ml	19	2	3	

Finally, the current study revealed that 62 (43.35%) patients from both groups were normal for depression, whereas 101 (70.62%) participants were normal for anxiety. Approximately, 28 (19.58%) and 16 (11.18%) participants for depression and anxiety, respectively, were found at the borderline level. Similarly, 53 (37.06%) and 26 (18.18%) participants were found to have depression and anxiety, respectively (Table 5).

**Table 5:** The relative incidence of depression and anxiety

Patient group	No. of participants	Depression (%)			Anxiety (%)		
		Normal	Borderline	Abnormal	Normal	Borderline	Abnormal
Control Group	81	46 (56.70)	16 (19.75)	19 (23.45)	65 (80.24)	7 (8.64)	9 (11.11)
Study Group	62	16 (25.80)	12 (19.35)	34 (54.83)	36 (58.06)	9 (14.51)	17 (27.41)
Total	143	62 (43.35)	28 (19.58)	53 (37.06)	101 (70.62)	16 (11.18)	26 (18.18))

## Discussion

This study confirmed the association of hyperparathyroidism with depression and anxiety among chronic hemodialysis patients in the Al Baha Region of the Kingdom of Saudi Arabia. We studied the effect of gender as a risk factor and the incidence of depression and anxiety among this population. Hyperparathyroidism was defined in the study group as PTH >400 pg/ml. This level is selected following Kidney Disease Outcomes Quality Initiative (KDOQI) guidelines, which recommend a PTH level between 150 pg/ml and 300 pg/ml. This PTH level is two to nine times the upper limit of normal per KDIGO guidelines. Any level of more than 400 pg/ml was associated with increased mortality [15-17].

Elevated PTH levels in dialysis patients have been consistently linked to heightened levels of depression and anxiety, significantly impacting the patient’s quality of life [18,19]. Our findings align with existing research that indicates the association between depression and anxiety and iPTH levels, highlighting a potential correlation between these psychological factors and PTH levels in dialysis patients. In this article, we reported the association of a demographic feature, namely, gender, and laboratory result PTH level with increased risk of depression and anxiety. Moreover, older hemodialysis patients with elevated PTH levels exhibit a high prevalence of depression in many studies, indicating a negative impact on various aspects of their well-being [5]. We included patients from different age groups, though the largest population in our study comprised middle-aged patients. As a result, the estimated prevalence of depression remains high, indicating the need for further studies in the future to identify other demographic risk factors.

Conversely, patients on hemodialysis with low PTH levels show a high proportion of normal HADS scores for anxiety and depression. This result underscores the potential correlation between PTH levels and mental health in individuals undergoing hemodialysis. Our study suggests that maintaining optimal PTH levels may play a crucial role in mitigating anxiety and depression among hemodialysis patients [20,21].

The prevalence of CKD worldwide is approximately 13.4%, with up to seven million patients receiving dialysis. This situation raises calls for research on the factors associated with increased morbidity in these populations, such as depression and anxiety [22]. Gender is one of the critical factors searched in many studies. A systematic review found that female patients in the general population are more prone to anxiety than males, resulting from psychosocial and biological factors [23]. Among the hemodialysis population, a systematic review found that anxiety prevalence has no significant difference compared with the general population and raised concern about the role of PTH level [24]. In the current study, gender differences in anxiety levels were consistent with existing literature. Thus, women can be said to be more prone to anxiety disorders compared with men, particularly in the group with PTH >400 pg/ml. This finding indicates that further studies in the future are needed to explore the impact of different abnormal laboratory results on increased risk of anxiety.

The observed gender differences in depression among hemodialysis patients from previous studies revealed that women exhibiting high depression scores have a lower quality of life compared with men [25,26]. Another study found that males have a higher prevalence than females [27], but no study correlated their result to PTH levels as in the current study. The reason is that they raised an essential concern that pain and lack of sleep, which are the most common symptoms of elevated PTH, can lead to depression [28]. This study estimated that male patients have a significant association with depression when compared with female patients, suggesting the need for a nuanced approach to addressing mental health disparities among hemodialysis patients.

Similarly, the findings on the potential link between high PTH levels and an increased prevalence of abnormal depression scores in hemodialysis patients align with existing research associating excess PTH with neuropsychiatric disturbances [5]. Diabetes and hypertension were the most common primary cause of end-stage renal disease among participants in this study. Patients diagnosed with diabetes mellitus are prone to major depression, and the disease is associated with elevated PTH and linked to bone disease [29,30]. Further studies are required to understand this relation.

The study limitations include a small sample size and the use of a self-reported questionnaire (HADS) that could have a recall bias. In-depth interviews and a review of medical records to verify response accuracy were conducted to minimize these limitations. The study’s strength involved study and control groups for comparison. Additionally, the association between demographic and laboratory factors, such as PTH and gender, was explored, given that only a few studies have investigated these two factors simultaneously.

Due to the negative impact of these mental disorders on daily life and overall well-being, further research is required to elucidate the underlying mechanisms of the relationship between elevated PTH levels and depression and anxiety in hemodialysis patients.

## Conclusions

This study supports and advances the evidence of the association between hyperparathyroidism and mental health outcomes in chronic hemodialysis patients. The observed gender differences underscore the need for tailored interventions to address anxiety and depression in this population. The study emphasizes the importance of routine mental health assessments, utilizing tools such as the HADS, in the comprehensive care of hemodialysis patients. Future research should focus on elucidating the underlying demographic features and potential therapeutic strategies to improve mental health outcomes in hemodialysis patients.
